# Hydrogen Sulfide Releasing 2-Mercaptoacrylic Acid-Based Derivative Possesses Cytoprotective Activity in a Small Intestine of Rats with Medication-Induced Enteropathy

**DOI:** 10.3390/scipharm85040035

**Published:** 2017-10-24

**Authors:** Yulia Sklyarova, Iryna Fomenko, Iryna Lozynska, Andrii Lozynskyi, Roman Lesyk, Alexandr Sklyarov

**Affiliations:** 1 Department of Biochemistry, Danylo Halytsky Lviv National Medical University, Lviv 79010, Ukraine; meriy7777777@gmail.com (Y.S.); ira9ilkiv@gmail.com (I.L.); o.y.sklyarov@gmail.com (A.S.); 2 Department of Pharmaceutical, Organic and Bioorganic Chemistry of Danylo Halytsky Lviv National Medical University, Lviv 79010, Ukraine; lozisnskij@i.ua (A.L.); dr_r_lesyk@org.lviv.net (R.L.)

**Keywords:** enteropathy, hydrogen sulfide, 2-mercaptoacrylic acids, small intestine

## Abstract

Small intestinal injury is known to be one of the most commonly appearing pathologies, resulting in the use of medications such as: nonsteroidal anti-inflammatory drugs (NSAIDs), antitumor drugs and angiotensin-converting enzyme (ACE) inhibitors. The principal objective of this study is to evaluate the action of a novel mercaptoacrylic acid derivative able to release H_2_S on parameters of NO-synthase system and oxidative stress. Inducing enteropathy, three types of medications were used: indomethacin, an NSAID (35 mg/kg); methotrexate, an antitumor drug (10 mg/kg); and enalapril, an ACE inhibitor (2 mg/kg/day). 2-[(4-chlorophenyl-carbamoyl)-methyl]-3-(3,5-di-tert-butyl-4-hydroxyphenyl)-acrylic acid (2C3DHTA) was introduced based on the background of medication-induced enteropathy (10 mg/kg/day). The survey showed that malondialdehyde (MDA) concentration, myeloperoxidase (MPO) activity, superoxide dismutase (SOD), catalase, and NO-synthases (NOS) were determined in the small intestinal mucosa. The increase in inducible NO-synthase (iNOS) activity was due to indomethacin and methotrexate administration. Constitutive NO-synthase (cNOS) activity was decreased by an ACE-inhibitor. The cytoprotective effect was demonstrated by 2C3DHTA, which returned iNOS activity to its control level and increased cNOS activity. The enterotoxic action of studied medication was accompanied by the development of oxidative stress manifested, activity of MPO was increased. MPO activity and manifestations of oxidative stress were decreased by 2C3DHTA. Effects of 2C3DHTA can be explained by the action of H_2_S, released from this compound in the gastrointestinal (GI) system.

## 1. Introduction

The development of various medical therapies has led to an increased frequency of medication side-effects [1]. To our knowledge, drug-induced injury most commonly affects gastrointestinal and hepatobiliary systems on account of mechanisms of drug absorption and metabolism [1,2]. Small intestinal injury is one of the most commonly appearing pathologies, leading to the development of enteritis (acute or chronic inflammation of small intestine). 

Among many medications, non-steroidal anti-inflammatory drugs (NSAIDs), antitumor drugs and hypotensive medications that inhibit the activity of angiotensin-converting enzyme (ACE) possess the highest enterotoxicity and influence the status of small intestinal microbiota [3]. 

NSAIDs are the main offenders in drug-induced injuries [3]. The pathogenesis of NSAID-enteropathy is complex. On one hand, NSAIDs are weak and lipophilic acids cause so-called topic effects in the small intestine by disrupting the phospholipid membranes of enterocytes and causing uncoupling of oxidative phosphorylation in mitochondria. This leads to the breakdown of the mucosal barrier and cellular death by apoptosis/necrosis, resulting in increased intestinal permeability and exposing the epithelium to bile acids, pancreatic secretions, and intestinal bacteria, resulting in inflammation [1,4]. On the other hand, NSAIDs inhibit prostaglandins (PGs) production by cyclooxygenase (COX). This inhibition renders the small intestine much more susceptible to damage induced by luminal irritants and makes it less able to withstand and restore mucosal structure and function after injury [5].

Furthermore, chemotherapeutic agents also cause serious injury to the small intestine. In particular, methotrexate damages the small intestinal mucosa [6] by preventing crypt mitotic activity, inhibiting dihydrofolate reductase and subsequently impairing the absorption of folic acid as well as d-xylose, and leading to the development of malabsorption syndrome and diarrhoea [7]. Similar morphologic changes in the small intestine have been noted with 5-fluorouracil treatment for malignant disorders in some patients [6]. Other possible mechanisms of antitumor drugs’ injuring effect in the small intestine is pneumatosis intestinalis, a condition in which gas is present in the wall of the small bowel. One proposed mechanism of drug-induced pneumatosis is a loss of mucosal integrity, which allows intraluminal gas to escape into the intestinal wall [1].

In addition, ACE inhibitors are the most widely used drugs for the treatment of hypertension. These medications inhibit the breakdown of bradykinin [8]. Bradykinin subsequently activates the nitric oxide (NO) system, leading to increased vascular permeability and capillary leakage. Thus, ACE inhibitors induce bowel angioedema, manifested in patients as abrupt-onset abdominal pain and nausea, with vomiting and sometimes diarrhoea [1].

Taking into consideration the fact that NSAIDs, ACE inhibitors and antitumor drugs are widely used for the treatment of various serious diseases, the search for new drugs without any side effects is an important medical and pharmaceutical problem. A new approach in this sphere may be demonstrated by novel sulfur-containing compounds based on mercaptoacrylic acids and thiazolidinones as their synthetic precursors possessing a dual COX/lipoxygenase (LOX) inhibitory action. In addition, thiazolidinones and their structure-related derivatives are an important class of organosulfur compounds with a diverse range of pharmacological activities such as anticancer [9], antioxidant [10], anti-inflammatory [11,12], antimicrobial [13]. One of the most potent dual COX/LOX inhibitors based on 4-thiazolidinone derivatives is darbufelone (2-amino-5-(3,5-ditertbutyl-4-hydroxybenzylidene)-thiazol-4-one) [14,15]. Chemical insertion of a H_2_S-donating moiety into biologically relevant molecules demonstrated cytoprotective action in pilot studies [16,17]. 

H_2_S has been proposed as the third gasotransmitter, in addition to nitric oxide (NO) and carbon monoxide (CO), and is involved in inflammation, intestinal motility, oxidative stress, ulcer healing, vascular tone, neuromodulation, apoptosis and many other vital biological functions [18]. H_2_S was found in many tissues, including the gastrointestinal (GI) tract, which is a major site of H_2_S production. Additionally, there is much evidence demonstrating beneficial effects of H_2_S-donating medications in the GI tract [18,19]. Among many effects, H_2_S-releasing compounds can influence NO-synthase system. It is well known that NO is a signaling molecule that plays a key role in the pathogenesis of inflammation. It possesses an anti-inflammatory activity under normal physiological conditions. On the other hand, NO is considered as a pro-inflammatory mediator that induces inflammation due to overproduction in abnormal situations. NO is synthesized and released by the assistance of NO-synthases that convert arginine into citrulline producing NO [20]. Constitutive NO-synthase (cNOS) (endothelial and neuronal) are Ca^2+^-dependant enzymes responsible for physiological functions of NO in a small intestine, and inducible NO-synthase (iNOS) is considered to be a pro-inflammatory enzyme [21].

The purpose of this study is to evaluate the action of a novel 2-mercaptoacrylic acid-based derivative, possessing dual COX/LOX inhibitory action and able to release H_2_S on parameters of NO-synthase system and oxidative stress under conditions of physiological norms and based on the background of drug-induced enteropathy. 

## 2. Methods

### 2.1. Animals

The structure of this study and the experimental procedures performed on the animals were approved by the Ethical Committee of Lviv National Medical University (Protocol N2 dated by 16 February 2015). The experimental procedures were carried out in accordance with the international guidelines for the use and care of laboratory animals. Male, outbred albino rats weighing 200–220 g were used. They were group housed. The rats were housed under condition of controlled temperature (21–22 °C), humidity (65–75%) and light cycle (12 h light/12 h dark) and fed standard rat chow and water ad libitum. 

### 2.2. Models of Medication-Induced Enteropathies

Three types of medications were used to induce enteropathy: indomethacin, an NSAID was introduced in a single dose of 35 mg/kg subcutaneously as previously described [16]; metothrexate, an anti-tumour drug was introduced in a single dose of 10 mg/kg, intraperitoneally [22]; enalapril, an ACE inhibitor was introduced three times in a dose 2 mg/kg/day [23] three days after the introduction of indomethacin or methotrexate and on the third day after daily introduction of enalapril enteropathy had developed.

### 2.3. Test Drugs

**Figure 1:**
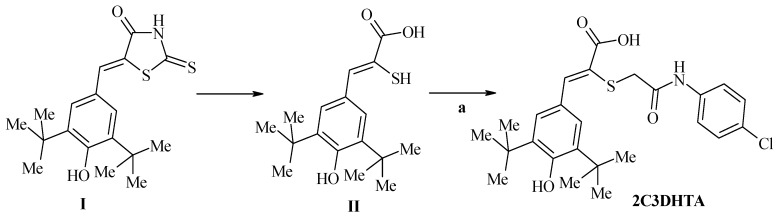
Synthesis of 2-[(4-chlor-phenyl-carbamoyl)-methyl]-3-(3,5-di-tert-butyl-4-hydroxyphenyl)-acrylic acid (2C3DHTA). Reagents, conditions and yields: 2,4′-dichloroacetanilide, EtOH, reflux 3 h, 72%.

The COX/LOX dual inhibitor darbufelone (2A5DHT) and the novel 2-mercaptoacrylic acid-based derivative, a compound 2-[(4-chlorophenyl-carbamoyl)-methyl]-3-(3,5-di-tert-butyl-4-hydroxyphenyl)-acrylic acid (2C3DHTA) were synthesized by Prof. Roman Lesykin the Department of Pharmaсeutical, Organic and Bioorganic Chemistry at Danylo Halytsky Lviv National Medical University (Figure 1). Synthesis of 2A5DHT was described previously [15]. 2C3DHTA, being an anti-inflammatory compound, possesses the dual COX/LOX inhibitory activity and potentially releases H_2_S in digestive system. 

The starting 5-(3,5-di-tert-butyl-4-hydroxybenzylidene)-2-thioxothiazolidin-4-one (**I**) and 3-(3,5-di-tert-butyl-4-hydroxyphenyl)-2-mercaptoacrylic acid (**II**) were obtained according to method described previously [16]. 

The elemental analyses were performed using the PerkinElmer 2400 CHN analyzer (Waltham, MA, USA). Analyses indicated by the symbols of the elements or functions were within ±0.4% of the theoretical values. The ^1^H NMR spectra was recorded on a Varian Gemini 400 MHz instrument (Varian Medical Systems, Palo Alto, CA, USA) in DMSO-*d*_6_. Chemical shifts (δ) are given in ppm units relative to tetramethylsilane as reference (0.00). 

To a stirred suspension of 3-(3,5-di-tert-butyl-4-hydroxyphenyl)-2-mercaptoacrylic acid (**II**) (5 mmol) in 30 mL of ethanol was added 15 mL ethanol solution of potassium hydroxide (10 mmol). After the reaction mixture was stirred at room temperature for 1 h, to the resulting potassium salt was added 2-chloro-*N*-(4-chloro-phenyl)-acetamide (5 mmol) and reaction mixture was refluxed for 3 h. The obtained precipitate was filtered and washed with methanol and diethyl ether and recrystallized from ethanol.

Yield 72%, ^1^H NMR (400 MHz, DMSO-*d*_6_): δ 1.43 (s, 18Н, *t-*Bu), 3.51 (s, 2H, CH_2_), 7.37 (d, 2Н, *J* = 8.8 Hz, arom.), 7.61 (d, 2Н, *J* = 8.8 Hz, arom), 7.70 (s, 2H, arom.), 7.72 (s, 1Н, CH), 8.03 (s, 1Н, OH), 9.84 (s, 1Н, NH), 10.28 (br.s, 1Н, COOH). Anal. Calcd. for C_25_H_30_ClNO_4_S: C, 63.08; H, 6.35; N, 2.94. Found: C, 63.19; H, 6.21; N, 2.81.

### 2.4. Study Protocol

The study was divided into two stages: (1) evaluation of effects of dual COX/LOX inhibitors in the small intestine of rats under condition of physiological norm; and (2) determination of 2C3DHTA action in the small intestine based on the background of drug-induced enteropathy (Table 1). 

According to the design of the study the rats were randomly divided into 9 groups: 1—rats from a control group; 2—animals that received 2A5DHT; 3—rats that were treated with 2C3DHTA: 4—enteropathy that was induced by indomethacin; 5–animals that received 2C3DHTA based on the background of indomethacin-induced injury; 6—enteropathy that was induced by methotrexate; 7—rats that received 2C3DHTA based on the background of methotrexate-induced enteropathy; 8—enteropathy induced by enalapril; 9—animals that received 2C3DHTA based on the background of enalapril-induced enteropathy. Before administration, the studied compounds (2C3DHTA and 2A5DHT) were dissolved in a small amount of DMSO, then suspended in 1% carboxymetylcellulose. Animals of the 3rd, 5th, 7th and 9th groups received 2C3DHTA in a dose of 10 mg/kg/day intraperitoneally once daily per 24 h (the first time 30 min before induction of enteropathy), and animals of 1st, 4th, 6th and 8th groups received the vehicle.

Rats were anesthetized with 1 mL of urethane at a dose of 1.1 mg/kg injected intraperitoneally and sacrificed by cervical dislocation. A blood sample from the cervical vessel was immediately collected into vials containing 0.1 mL of heparin. The samples of small intestinal mucosa were homogenised in phosphate buffer pH 6.0 1:4 and centrifuged at 1500 g. Supernatant was used to determine values of biochemical parameters.

### 2.5. Biochemical Assessment

*Determination of NO-system parameters in small intestinal mucosa.* Activity of NO-synthase isoenzymes (inducible iNOS and constitutive cNOS) was measured by the method described in detail [18]. NOS activity was expressed in nmol l-citrylline/min×mg of protein. NOx (nitrite/nitrate) concentration in homogenates of small intestinal mucosa was assayed by the Griess reaction-dependent method of [24]. In order to determine total (NO_2_/NO_3_) concentration to deproteinised homogenates (1:100) of zinc for reduction of nitrate to nitrite or manganese sulphate for measurement of nitrate-anion were added. Naphthyl-ethylenediamine was used to perform a Griess reaction [25]. The absorbance was read in a spectrophotometer Statfax at 520–560 (550) nm (Awareness Technology, Palm City, FL, USA). Concentration of stable products of NO was expressed as nitrite+nitrate (mmol/g).

*Measurement of l-arginine and H*_2_*S in blood serum.* The level of l-arginine in plasma samples was measured by the Sakaguchi reaction [26]. Plasma l-arginine level was expressed as mmol/L. 

H_2_S concentration was determined by reaction with para-phenylenediamine [27]. To 0.1 mL of blood serum, 0.5 mL of 1% zinc acetate, 0.5 mL of 50 mmol *p-*phenylenediamine and 0.4 mL of 30 mmol FeCl_3_ were added and incubated for 10 min. Then, 1.0 mL of 20% trichloroacetic acid was added to precipitate any protein that might be present in the media and centrifugation (10,000 *g*) was performed. Absorbance (540 nm) of aliquots from the resulting supernatant was determined. The calibration curve of absorbance vs. H_2_S concentration was obtained by using the NaHS solution.

Myeloperoxidase (MPO) activity in homogenates of small intestinal mucosa was assayed spectrophotometrically by the method of [28] with some modifications. The MPO activity was analysed spectrophotometrically as follows: 1 mL of homogenate was added to 2.9 mL of 0.1 M K_3_PO_4_ buffer (pH 6.0) involving *o*-dianisidine dihydrochloride (0.167 mg/mL) and 0.005% hydrogen peroxide of the resection mixture was recorded at a wave length of 450 nm. One unit (U) of activity was defined as that degrading 1 µmole of peroxide/mg of protein.

*Lipid peroxidation.* Levels were determined as malondialdehyde (MDA) concentration in homogenates of small intestinal mucosa, according to the procedure of [29]. MDA levels were expressed as mmol/L.

*Activity of antioxidant enzymes defence determination.* The activity of superoxide dismutase (SOD) was determined by the reaction of reduction of nitrotetrazolium blue to nitroformazan [30]. SOD activity was expressed in mmol/min × mg of protein. Catalase (CAT) activity was determined by measuring of the decrease in hydrogen peroxide concentration at 410 nm [31]. Catalase activity was expressed in mmol H_2_O_2_/min × mg of protein. 

### 2.6. Statistics

The statistical processing of the data was done by conventional methods for analysis of variance using MS Excel software for Student’s *t*-test. The difference was considered to be significant at *p* ≤ 0.05.

## 3. Results

**Figure 2:**
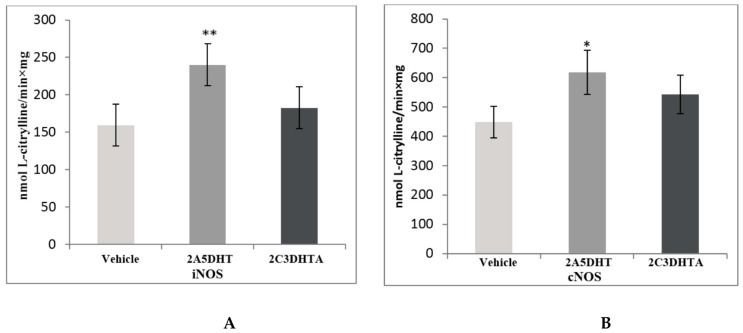
Activity of nitric oxide synthases (iNOS—(**A**) and cNOS—(**B**)) in homogenates of small intestinal mucosa of rats of the following groups: group 1—control group (vehicle), group 2—action of a dual COX/LOX inhibitor—2A5DHT, group 3—action of H_2_S releasing dual COX/LOX inhibitor—2C3DHTA. Mean ± SD, *n* = 8 in each group of animals. * *p* ≤ 0.05, ** *p* ≤ 0.01, in relation to control animals.

Administration of both studied dual COX/LOX inhibitors (2A5DHT and 2C3DHTA) did not induce any visible ulcerative lesion of small intestinal mucosa. 2A5DHT caused an increase of both NO-synthase isoenzymes (iNOS and cNOS) by 51%, *p* ≤ 0.01 and 38%, *p* ≤ 0.05, correspondently (Figure 2). 2C3DHTA caused only the tendency to their increase. 

**Figure 3:**
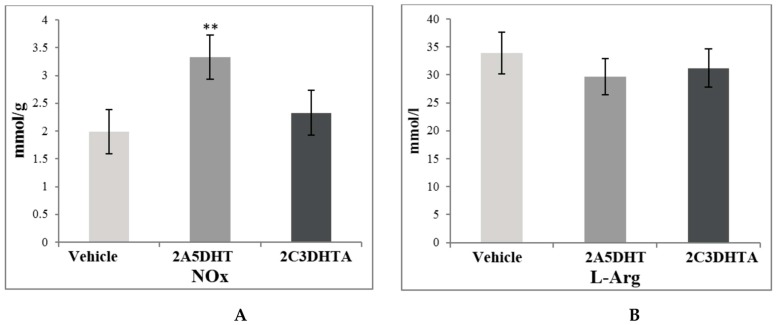
The concentrations of stable products of NO (NOx) in homogenates of small intestinal mucosa (**A**) and the level of l-Arginine (l-Arg) in blood serum (**B**) of rats of the following groups: group 1—control group (vehicle), group 2—action of a dual COX/LOX inhibitor—2A5DHT, group 3—action of H_2_S releasing dual COX/LOX inhibitor—2C3DHTA. Mean ± SD, *n* = 8 in each group of animals. ** *p* ≤ 0.01, in relation to control animals.

In correspondence to the increase of NO-synthases activity, the concentration of NO_x_ increased in the group of animals treated by 2A5DHT by 67%, *p* ≤ 0.01, and it did not change after 2C3DHTA administration (Figure 3). The changes of l-Arginine concentration in blood plasma in studied groups were not statistically significant. 

Administration of dual COX/LOX inhibitor 2A5DHT resulted in the tendency in the decrease of H_2_S concentration in blood plasma, the increase in MPO activity by 50%, *p* ≤ 0.01 and MDA concentration by 15%, *p* ≤ 0.01 (Table 2). 2C3DHTA being a derivative of 2A5DHT did not decrease H_2_S level because of its ability to release H_2_S in the GI tract. However, it also increases MDA concentration similarly to its parent compound 2A5DHT indicating activation of the lipid peroxydation process.

Thus, we can conclude, that H_2_S-releasing dual COX/LOX inhibitor 2C3DHTA didn’t cause any serious changes in NO-system parameters and MPO activity as compared to its parent compound 2A5DHT. It maintained physiological level of H_2_S concentration in blood plasma but increased MDA concentration.

In the second stage of the study, we modulated drug-induced small intestinal injury. Administration of indomethacin induced the development of ulcerative lesion of small intestinal mucosa manifested by erosions and haemorrhages, localised mainly in the distal part of the small intestine in correspondence to previously described results [16]. Neither metothrexate nor enalapril caused any visible changes of the small intestine surface. It should be pointed out that metothrexate-treated animals were suffering from severe enterotoxicosis manifested by diarrhoea and vomiting. 

**Figure 4:**
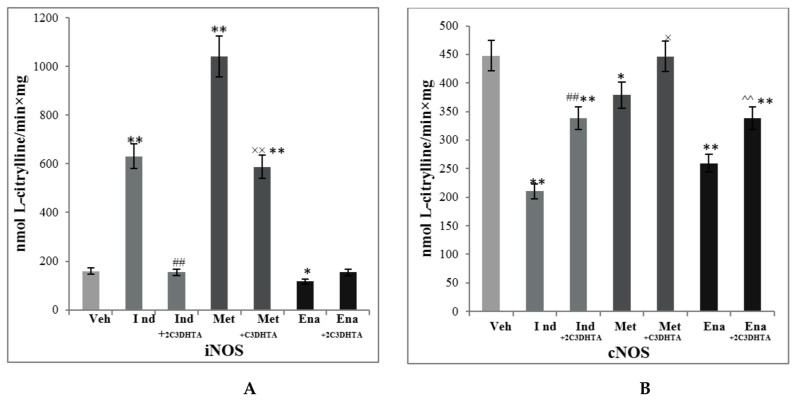
Effect of 2C3DHTA activity of nitric oxide synthases (iNOS—(**A**) and cNOS—(**B**)) in homogenates of small intestinal mucosa at the background of drug-induced enteropathy of rats of the following groups: group 1—control group (Veh), group 4—indomethacin-induced enteropathy (Ind), group 5—indomethacin + 2C3DHTA (Ind + 2C3DHTA), group 6—metothrexate-induced enteropathy (Met), group 7—metothrexate + 2C3DHTA (Met + 2C3DHTA), group 8—enalapril-induced enteropathy (Ena), group 9—enalapril + 2C3DHTA (Ena + 2C3DHTA). Mean ± SD, *n* = 8 in each group of animals. * *p* ≤ 0.05, ** *p* ≤ 0.01, in relation to control animals; ^##^ *p* ≤ 0.01 as compared to the indomethacin action; ^×^ *p* ≤ 0.05, ^××^ *p* ≤ 0.01, as compared to metothrexate action; ^^^^ *p* ≤ 0.01, as compared to enalapril action.

In spite of different mechanisms of their action upon metabolic processes in the small intestine, all used medications (indomethacin, metothrexate and enelapril) caused changes of NO-synthase system parameters. In our study, administration of indomethacin, metothrexate caused a rise in iNOS activity (almost fourfold and more than fivefold correspondently (*p* ≤ 0.01)) (Figure 4). Simultaneously cNOS activity decreased more than twofold in indomethacin-treated group (*p* ≤ 0.01) and by 15% in metothrexate-treated group (*p* ≤ 0.05). The ACE-inhibitor practically didn’t influence iNOS, but it still decreased cNOS activity almost twofold (*p* ≤ 0.01). 

Administration of a novel COX/LOX inhibitor, an H_2_S releasing compound 2C3DHTA demonstrated a significant cytoprotective effect in all used models of drug-induced enteropathies. It returned iNOS activity to its control level and increased cNOS activity by 60% (*p* ≤ 0.01) in the group of its simultaneous action with indomethacin. In the group of metothrexate-treated rats, 2C3DHTA decreased iNOS activity by 56% and returned cNOS activity to its control level (*p* ≤ 0.01). 2C3DHTA didn’t demonstrate any significant influence on iNOS activity; however, it increased cNOS activity by 30% for the group of enalapril-induced enteropathy.

**Figure 5:**
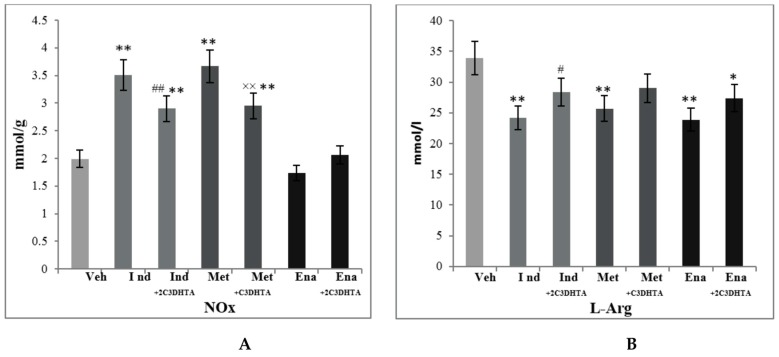
Concentrations of stable products of NO in homogenates of small intestinal mucosa (**A**) and the level of l-Arginine (l-Arg) in blood serum (**B**) at the background of drug-induced enteropathy of rats of the following groups: group 1—control group (Veh), group 4—indomethacin-induced enteropathy (Ind), group 5—indomethacin + 2C3DHTA (Ind + 2C3DHTA, group 6—metothrexate-induced enteropathy (Met), group 7—metothrexate + 2C3DHTA (Met + 2C3DHTA), group 8—enalapril-induced enteropathy (Ena), group 9—enalapril + 2C3DHTA (Ena + 2C3DHTA). Mean ± SD, *n* = 8 in each group of animals. * *p* ≤ 0.05, ** *p* ≤ 0.01, in relation to control animals; ^#^ *p* ≤ 0.05, ^##^ *p* ≤ 0.01 as compared to the indomethacin action.

In correspondence to changes in NO-synthases activity, NO concentration significantly varied in studied groups: it was markedly elevated in indomethacin- and metothrexate-induced enteropathies by 76% and 84% (*р* ≤ 0.01) concomitantly and decreased by 13% (*p* ≤ 0.05) in a group of enalapril-treated rats (Figure 4). Introduction of 2C3DHTA at the background of drug-induced enteropathies decreased NO concentration as compared to indomethacin and metothrexate action and increased it as compared to enalapril administration. l-Arginine is a precursor for NO synthesis in mammals. Thus, the changes in NO-synthases activity and stabile NO products in homogenates of small intestinal mucosa (MMSI) led to a decrease of l-Arginine concentration in blood serum in indomethacin- and metothrexate-treated rats, indicating its increased utilisation for the synthesis of NO in the small intestine (Figure 5). 

In spite of the decrease of both cNOS activity and NO concentration in the group of enalapril-treated rats, the l-Arinine level was significantly lower (by 30%, *p* ≤ 0.01) than in rats of the control group. 2C3DHTA administration demonstrated the tendency to normalisation of l-Arginine concentration in all studied groups. 

H_2_S concentration in blood was decreased in all models of drug-induced enteropathies, which can be accompanied by the loss of gastrointestinal mucosal resistance to injury. H_2_S-releasing compound 2C3DHTA elevated H_2_S level in blood serum of rats with drug-induced enteropathies (Table 3).

Enterotoxic action of studied medication was accompanied by the development of oxidative stress in small intestinal mucosa manifested by the rise of MDA concentration and the significant increase of antioxidant enzymes (SOD and CAT). In addition, activity of MPO was increased as compared to the control group. H_2_S-releasing 2-mercaptoacrylic acid-based derivative 2C3DHTA decreased MPO activity and manifestations of oxidative stress. However, in the group of indomethacin-induced enteropathy, 2C3DHTA didn’t influence MDA concentration and SOD, CAT activities. This can be result in double COX inhibition (by indomethacin and 2C3DHTA) and the loss of cytoprotective activity of PGs.

## 4. Discussion

In the last few years, numerous studies have demonstrated the roles of H_2_S in physiology and disease. H_2_S has been found to have dichotomous effects (stimulatory and inhibitory) on several gastrointestinal processes such as inflammation, contractile responses, nociception, cancer and apoptosis. H_2_S has been found to be protective in several animal models of injury in the gastrointestinal tract, brain, lung, kidney and heart [18]. In our study, we chose models of drug-induced injury of the small intestine. Drugs used to induce enteropathy belong to three different classes of medications: NSAIDs, anti-tumor and hypotensive drugs. All of them are known to subsequently affect H_2_S synthesis in the GI tract. This fact served as the theoretical basis for the development of novel drug hybrids. In these hybrids, the chemical insertion of a H_2_S-donating moiety into established drugs results in new chemical entities with significant improvements in activity and/or safety [32]. In vivo, the novel compounds entity releases the parent compound, which acts according to their own pharmacological properties, while the slowly released H_2_S has marked activity in inflammatory cells and a cytoprotective effect in noninflamed tissue [32]. A range of NSAID derivatives that release H_2_S have been synthesised and evaluated, with consistent results in terms of retaining anti-inflammatory activity but reducing GI toxicity. For instance, derivatives of aspirin, naproxen, ketoprofen, indomethacin, ibuprofen, flurbiprofen and diclofenac were synthesised, each with a number of different H_2_S-releasing moieties [33]. In our study, a novel 2-mercaptoacrylic acid-based derivative—2C3DHTA—was used. This compound was synthesized on the basis of its parent compound 2-amino-5-(3,5-ditertbutyl-4-hydroxybenzylidene)-thiazol-4-one (2A5DHT), which is an active substance of a well known dual COX/LOX inhibitor darbufelone [14,15,34]. In a randomized, double blind, rising single-and multiple-dose study in healthy volunteers, it was shown that darbufelone was well tolerated up to the oral daily dose of 100 mg and demonstrated anti-inflammatory effects [14]. Dual COX/LOX inhibitors constitute a valuable alternative to classical NSAIDs and selective COX-2 inhibitors for the treatment of inflammatory diseases. Moreover, they appear to be almost exempted from gastrointestinal toxicity [15,34]. Chemical insertion of a H_2_S-donating moiety into establishing a parent compound resulted in its new properties. In pilot studies, such H_2_S-releasing COX/LOX inhibitors demonstrated cytoprotective activity manifested by reduction of nitroso-oxidative processes caused by administration of NSAIDs and acute stress in the small intestine. [16,17]. Thus, this study was designed on one hand to evaluate anti-inflammatory action of a novel 2-mercaptoacrylic acid-based derivative as analogues of biologically active 4-thiazolidinones and, on the other hand, to show a beneficial effect of this drug based on the background of drug-induced enteropathies. 

H_2_S-releasing dual COX/LOX inhibitor 2C3DHTA didn’t possess any ulcerogenic action in noninflamed small intestine as compared to COX inhibitor indometacine. It should be pointed out that it didn’t induce the activity of pro-inflammatory enzymes (iNOS and MPO) unlike its parent compound 2A5DHT. Still it increased MDA concentration, we suppose that it may be a result of the loss of cytoprotective prostaglandins and leukotrienes. 

Alterations of biochemical parameters obtained in this study in different models of drug-induced enteropathies are similar to previously described results [7,16] manifested by formation of nitroso-oxidative stress. Administration of a substituted 2-mercaptoacrylic acid derivative led to normalization of NO-synthases activities and the intensity of lipid peroxidation processes in homogenates of small intestinal mucosa. It was resulted mainly by H_2_S action, released from 2C3DHTA. It was previously shown that H_2_S interacts on redox imbalance processes, such as reactive oxygen species (ROS) generation and transcription factor activation [32]. There is the relationship between NO and H_2_S in the GI tract, which is likely mediated through the regulation of genes expression [35,36]. It also recently was shown that H_2_S possesses chemopreventive activity [37].

Anti-inflammatory effects of 2C3DHTA shown in our study were manifested by the decrease of iNOS and the increase of cNOS activities, the decrease of MDA concentration and MPO activity, the increase of antioxidant defence enzymes SOD and CAT. We suppose that all these effects appeared as a result of both dual COX/LOX inhibition and the release of H_2_S. Dual COX/LOX inhibition in group of indomethacin-induced enteropathy didn’t reduce the manifestation of oxidative stress. Probably it was a result of a double COX inhibition (by indomethacin and 2C3DHTA) and the loss of cytoprotective PGs, involved in defense of small intestine. However even under condition of double COX inhibition, 2C3DHTA displayed its cytoprotective effects. 

## 5. Conclusions

A novel 2-mercaptoacrylic acid-based derivative 2C3DHTA demonstrated the cytoprotective activity in the small intestine of rats with drug-induced enteropathy manifested by the reduction of oxidative stress, the decrease of the activity of pro-inflammatory enzymes myeloperoxydase and iNOS. Effects of 2C3DHTA on one hand can be explained by the action of H_2_S, released from this compound in the gastrointestinal system, and on the other hand by the dual inhibition of pro-inflammatory enzymes COX and LOX. Thus, in our study, we showed that H_2_S released from compound 2C3DHTA was involved in mechanisms’ cytoprotection in a small intestine. 

## Figures and Tables

**Table 1:** Study design.

**Stage 1. Evaluation of effects of dual COX/LOX inhibitors in the small intestine of rats under condition of physiological norm (in noninflamed mucosa)**					
Control group		Action of dual COX/LOX inhibitor		Action of H_2_S releasing COX/LOX inhibitor	
Vehicle		10 mg/kg/day 2A5DHT intraperitoneally		10 mg/kg/day 2C3DHTA intraperitoneally	
(*n* = 8)		(*n* = 6)		(*n* = 8)	
Group 1		Group 2		Group 3	
**Stage 2. Determination of 2C3DHTA action in the small intestine based on the background of drug-induced enteropathy**					
Indomethacin 35 mg/kg subcutaneously	Indomethacin 35 mg/kg subcutaneously	Metothrexate 10 mg/kg intraperitoneally	Metothrexate 10 mg/kg Intraperitoneally	Enalapril 2 mg/kg/day intraperitoneally	Enalapril 2 mg/kg/day intraperitoneally
Vehicle	10 mg/kg/day 2C3DHTA intraperitoneally	Vehicle	10 mg/kg/day 2C3DHTA intraperitoneally	Vehicle	10 mg/kg/day 2C3DHTA intraperitoneally
(*n* = 8)	(*n* = 8)	(*n* = 8)	(*n* = 8)	(*n* = 8)	(*n* = 8)
Group 4	Group 5	Group 6	Group 7	Group 8	Group 9

COX: cyclooxygenase; LOX: lipoxygenase; 2A5DHT: darbufelone active substance.

**Table 2:** The concentration of H_2_S in blood and activity of myeloperoxidase (MPO) and antioxidant enzymes (superoxide dismutase (SOD) and catalase (CAT)) and concentration of malondialdehyde (MDA) in small intestinal mucosa of rats treated by COX/LOX inhibitors. Mean ± SD, *n* = 8 in each group of animals.

Experimental Groups	Н_2_S (µmol/L)	MPO (U/mg)	MDA (µmol/g)	SOD (mmol/min × mg)	CAT (mmol H_2_O_2_/min × mg)
Control group	72.37 ± 2.48	1.49 ± 0.43	191.2 ± 32.7	25.63 ± 1.71	35.97 ± 2.48
2A5DHT	67.21 ± 2.53	2.24 ± 0.41 **	220.7 ± 18.9 **	23.35 ± 3.45	32.81 ± 3.41
2C3DHTA	75.36 ± 5.27	1.55 ± 0.35	236.2 ± 28.4 **	25.9 ± 2.39	30.29 ± 3.87

Note: ** *p* ≤ 0.01, in relation to control animals.

**Table 3:** The concentration of H_2_S in blood and activity of MPO and antioxidant enzymes (SOD and CAT) and concentration of MDA in small intestinal mucosa of rats with drug-induced enteropathies. Mean ± SD, *n* = 8 in each group of animals.

Experimental Groups	Н_2_S (µmol/L)	MPO (U/mg)	MDA (µmol/g)	SOD (mmol/min × mg)	CAT(mmol H_2_O_2_/min × mg)
Control group	72.37 ± 2.48	1.49 ± 0.43	191.2 ± 32.7	25.63 ± 1.71	35.97 ± 2.48
Indomethacin	60.22 ± 6.55 **	5.16 ± 0.98 **	268.2 ± 34.5 **	16.33 ± 2.45	17.78 ± 2.33 **
2C3DHTA + indomethacin	79.23 ± 8.58 ^##^	1.45 ± 0.75 ^##^	254.7 ± 29.65 **	19.4 ± 1.28 ^#^*	17.21 ± 1.99 **
Metothrexate	58.20 ± 5.41 **	2.18 ± 0.19 **	307.7 ± 52.1 **	14.18 ± 1.92 **	29.71 ± 1.11 **
2C3DHTA + metothrexate	69.34 ± 13.9	1.64 ± 0.84 ^×^	192.2 ± 40.43 ^××^	24.78 ± 4.1 ^××^	26.50 ± 4.35 **
Enalapril	69.61 ± 6.1	4.92 ± 1.73 **	250.4 ± 29.6 **	26.53 ± 2.41	26.08 ± 3.23 **
2C3DHTA + enalapril	73.8 ± 8,9	3.86 ± 0.89 ^^^**	218.0 ± 19.1 ^^^**	20.97 ± 3.02 ^^^**	24.99 ± 2.98 **

Note: * *p* ≤ 0.05, ** *p* ≤ 0.01, in relation to control animals; ^#^ *p* ≤ 0.05, ^##^ *p* ≤ 0.01 as compared to the indomethacin action; ^×^ *p* ≤ 0.05, ^××^ *p* ≤ 0.01, as compared to metothrexate action; ^^^ *p* ≤ 0.05, as compared to enalapril action.
